# Ligand‐Independent Actions of the Vitamin D Receptor: More Questions Than Answers

**DOI:** 10.1002/jbm4.10578

**Published:** 2021-11-23

**Authors:** Daniel D. Bikle

**Affiliations:** ^1^ Departments of Medicine and Dermatology University of California San Francisco, San Francisco VA Health Center San Francisco CA USA

**Keywords:** CALCITRIOL, CANCER, HAIR FOLLICLE, SKIN, VITAMIN D RECEPTOR

## Abstract

Our predominant understanding of the actions of vitamin D involve binding of its ligand, 1,25(OH)D, to the vitamin D receptor (VDR), which for its genomic actions binds to discrete regions of its target genes called vitamin D response elements. However, chromatin immunoprecipitation‐sequencing (ChIP‐seq) studies have observed that the VDR can bind to many sites in the genome without its ligand. The number of such sites and how much they coincide with sites that also bind the liganded VDR vary from cell to cell, with the keratinocyte from the skin having the greatest overlap and the intestinal epithelial cell having the least. What is the purpose of the unliganded VDR? In this review, I will focus on two clear examples in which the unliganded VDR plays a role. The best example is that of hair follicle cycling. Hair follicle cycling does not need 1,25(OH)_2_D, and Vdr lacking the ability to bind 1,25(OH)_2_D can restore hair follicle cycling in mice otherwise lacking Vdr. This is not true for other functions of VDR such as intestinal calcium transport. Tumor formation in the skin after UVB radiation or the application of chemical carcinogens also appears to be at least partially independent of 1,25(OH)_2_D in that *Vdr* null mice develop such tumors after these challenges, but mice lacking Cyp27b1, the enzyme producing 1,25(OH)_2_D, do not. Examples in other tissues emerge when studies comparing *Vdr* null and *Cyp27b1* null mice are compared, demonstrating a more severe phenotype with respect to bone mineral homeostasis in the *Cyp27b1* null mouse, suggesting a repressor function for VDR. This review will examine potential mechanisms for these ligand‐independent actions of VDR, but as the title indicates, there are more questions than answers with respect to this role of VDR. © 2021 The Author. *JBMR Plus* published by Wiley Periodicals LLC on behalf of American Society for Bone and Mineral Research.

## Graphical abstract

This model depicts the movement of the RXR/VDR from the cytoplasm into the nucleus, a step that can occur with or without 1,25(OH)_2_D, where it binds to a VDRE. When ligand bound, the RXR/VDR heterodimer activates genes promoting calcium transport, hormone secretion, and immune regulation, among other functions. In the absence of 1,25(OH)_2_D, the VDR/RXR heterodimer activates genes regulating hair follicle cycling and skin tumor suppression. This simplified model omits many of the processes underlying the mechanisms by which these events occur.

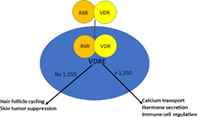

## Introduction

Nearly all known actions of the vitamin D receptor (VDR) involve its partnership with the active metabolite of vitamin D, 1,25(OH)_2_D. 1,25(OH)_2_D binds to VDR with high affinity and at least in its genomic actions drives the VDR into the nucleus, where it binds to specific regions in its target genes called vitamin D response elements (VDREs). This translocation of the VDR from the cytoplasm into the nucleus is highly regulated at least in part by retinoid X receptors (RXR) and is not necessarily ligand dependent.^(^ ^1^ ^)^ In particular, RXR, which forms a heterodimer with VDR in the cytoplasm, is capable of translocating VDR into the nucleus even in the absence of 1,25(OH)_2_D as long as the nuclear localization signal (NLS) of the RXR is intact. However, this translocation of the unliganded heterodimer is lost when the NLS of RXR is mutated but is regained with the addition of 1,25(OH)_2_D, which promotes the translocation of the RXR/VDR heterodimer regardless of whether the RXR NLS was intact as long as the NLS of the VDR is functional.^(^ ^1^, ^2^, ^3^ ^)^ But translocation into the nucleus is only half the story as the VDR can cycle in out of the nucleus again regulated by RXR, which reduces the export of the unliganded VDR from the nucleus but accelerates it in the presence of 1,25(OH)_2_D.^(^ ^1^ ^)^ That said, a significant number of sites in the genome appear to bind VDR in a persistent rather than transitory fashion.^(^ ^4^ ^)^

Analysis of data sets from several different cell types indicates more than 23,000 unique VDR binding loci in the human genome.^(^ ^4^ ^)^ 1,25(OH)_2_D increases VDR binding on average about ×2.5, but a substantial number of sites to which VDR bound do not require ligand.^(^ ^5^ ^)^ Of the ligand‐dependent VDR binding sites, most were direct repeats of two RGKTSA motifs (R = A or G, K = G or T, S = C or G) separated by three nucleotides (the classical DR3), whereas the sites to which ligand was not required for VDR binding were predominantly non‐classical motifs.^(^ ^5^ ^)^ Although the VDR is associated with a variety of complexes that alter its function, it is not clear that these complexes are distinctly different with respect to the type (classical or non‐classical) binding site involved. I will refer to both the classical and non‐classical VDR binding sites as VDREs. However, it should not be inferred that all VDREs so identified are regulatory in a given cellular context as well demonstrated when the VDREs of the*Tnfsf11* (Rankl) gene in cells of the mesenchymal and hematopoietic lineages are compared, showing that the VDREs regulating *Tnfsf11* expression differ between the two cell types.^(^ ^6^ ^)^ The extent to which ligand‐independent VDR binding occurs in a given genome is highly cell specific. For example, from chromatin immunoprecipitation studies of THP‐1 human monocytic leukemia cells, we learn that of the 1318 VDR binding sites, 789 showed an increase in VDR binding with 1,25(OH)_2_D administration, but 364 did not, and only 165 of these sites overlapped.^(^ ^5^ ^)^ In MC3T3E1 cells, VDR binding sites increased from 1325 to 8241 after 3 hours of 1,25(OH)_2_D administration.^(^ ^7^ ^)^ Even more extreme is the example of LS180 colon cancer cells in which VDR binding sites with vehicle alone numbered 262 but increased to 2209 with partial overlap after the addition of 1,25(OH)_2_D.^(^ ^8^ ^)^ On the other hand, in preliminary observations, we (Oda and colleagues) have found that 1,25(OH)_2_D has much less effect on VDR binding in the keratinocyte genome with 878 binding sites noted with vehicle, 929 sites after 1,25(OH)_2_D administration, and almost complete overlap. Although these numbers are dependent to a large extent on the experimental conditions of the analyses, they illustrate variability of ligand dependence/independence in different cell types. This raises the question of what function VDR is having on the genome in the absence of 1,25(OH)_2_D. Moreover, does the much greater percentage of ligand‐independent binding of VDR in the keratinocyte genome relative to the intestinal epithelial cell, osteoblast, or monocyte provide clues to the best characterized ligand‐independent actions of VDR—namely hair follicle cycling and resistance to UVB or chemically induced skin cancer? In this review, I will examine the role of VDR in hair follicle cycling and cancer resistance along with the concept that the unliganded VDR may regulate gene transcription in a manner different from what the liganded receptor would do. An important qualification is that throughout this review I will use the term unliganded VDR to mean independent of 1,25(OH)_2_D. There are other VDR ligands that may substitute for 1,25(OH)_2_D at least in some of its functions. Moreover, a full discussion of coregulators of VDR, both coactivators and corepressors, is outside the scope of this review, albeit quite important for the function of VDR.

## Hair Follicle Cycling

The hair follicle cycle is divided into three main stages: anagen, catagen, and telogen. The duration of these stages in a given species varies from location to location on the body and between sexes. Furthermore, there are two types of cycles: developmental and postnatal. The developmental cycle is initiated during embryogenesis. The follicle develops from specific regions of the epidermis called placodes. The development, number, and placement of these placodes are under the control of a number of factors but not VDR. The follicle is induced to grow by its interaction with a collection of specialized mesenchymal cells in the dermis called the dermal papilla. Wnt signaling (β‐catenin) appears to be necessary to maintain the ability of the dermal papilla to stimulate hair follicle growth.^(^ ^9^, ^10^ ^)^ After the developmental cycle, which leads to the initial coat of hair, the follicle undergoes repetitive cycling until senescence. Growth of the follicle occurs during anagen. The length of the hair is dependent on the duration of anagen. During this stage, the follicle grows through the dermis into the subcutaneous tissue. As the follicle develops, different cell layers appear. The outer root sheath (ORS) is a direct extension of the stratum basale, the basal layer of the epidermis, and separates the hair follicle from the surrounding connective tissue sheath (CTS). From outside in are found the companion layer, the three layers of the inner root sheath (IRS)—Henle's layer, Huxley's layer, cuticle of the IRS—and the hair shaft itself, including the cuticle of the shaft, shaft cortex, and shaft medulla. Stem cells in the bulge are capable of generating all cells in the hair follicle and epidermis,^(^ ^11^ ^)^ although stem cells reside in the stratum basale and infundibulum, where under normal circumstances they regulate growth and regeneration of the epidermis and sebaceous gland, respectively. These stem cells all express VDR. The keratins produced by the cells of the IRS and hair shaft differ from those expressed by epidermal keratinocytes.^(^ ^12^ ^)^ Of particular interest is these hair keratins have β‐catenin/lef1 binding sites in their promoters that regulate their expression.^(^ ^13^ ^)^ After anagen, the follicle enters catagen, during which massive apoptosis occurs primarily in the cells of the proximal follicle (the dermal portion), and the hair shaft produced during anagen is generally shed. The distal portion of the follicle (epidermal portion) remains intact during hair follicle cycling. At the end of catagen, the follicle enters telogen, the resting phase. A new cycle then begins with anagen. The juxtaposition of the dermal papilla to the bulge is critical for this process to begin, and it is associated with increased proliferation of stem cells in the bulge with migration of cells from the bulge into the hair bulb to restart the growth of the hair follicle. The regulatory elements that control the transition from one stage to the next are not well understood.

Alopecia is a well‐known part of the phenotype of many patients with mutations in their *VDR*,^(^ ^14^, ^15^ ^)^ a syndrome currently known as hereditary vitamin D‐resistant rickets (HVDRR). In that vitamin D deficiency per se is not associated with alopecia, the explanation for this phenomenon has remained obscure. Exploration of the link between alopecia and VDR received a major boost with the development of the *Vdr* null mouse by several groups.^(^ ^16^, ^17^, ^18^, ^19^ ^)^ These mice develop their first coat of hair normally, but reinitiation of anagen after the first cycle or after depilation is impaired.^(^ ^20^ ^)^ Reconstitution of the *Vdr* to the *Vdr* null mouse skin using a keratinocyte‐specific promoter reverses the defect in hair growth without reversing the metabolic defects of skeletal growth retardation, hypocalcemia, and rickets otherwise associated with the *Vdr* null condition.^(^ ^21^, ^22^ ^)^ Moreover, hair follicle cycling can even be restored when the *Vdr* is mutated in the ligand binding domain (L233S) or the AF2 domain (L417S) such that it no longer binds 1,25(OH)_2_D or is activated by it.^(^ ^23^ ^)^ Furthermore, inactivating mutations in *Cyp27b1*, the enzyme producing 1,25(OH)_2_D, do not result in alopecia, although the other metabolic defects (eg, rickets) found in *Vdr* knockout (KO) occur.^(^ ^24^, ^25^ ^)^ On the other hand, correction of the metabolic abnormalities with a high calcium rescue diet prevents the rickets and hyperparathyroidism but does not prevent the alopecia.^(^ ^26^ ^)^ Furthermore, it is the lack of VDR in the keratinocyte as opposed to the dermal papilla that is critical. Dermal papilla cells obtained from either *Vdr* null or wild‐type mice can initially induce hair growth in a hair reconstitution assay when mixed with epidermal keratinocytes obtained from wild‐type or *Vdr* null mice, but if the hair grown with keratinocytes from *Vdr* null mice is then depilated, anagen will not be reinitiated regardless of the source of dermal papilla cells.^(^ ^27^ ^)^

The mechanism mediating this ligand‐independent action of VDR is not fully understood. Although we know many genes and pathways that are ligand‐dependent actions of VDR in the skin and elsewhere, we know almost nothing about this ligand‐independent action. Clues come from the observation that the abnormality in hair follicle cycling in *Vdr* null mice (and humans) is essentially identical to that found in mice and humans with mutations in or null for hairless (*Hr*),^(^ ^28^, ^29^, ^30^, ^31^ ^)^ deletion of *Rxrα* and *Rxrβ*, transcriptional partners of Vdr,^(^ ^32^, ^33^ ^)^ mutations in *Ctnnb1* (β‐catenin)^(^ ^34^ ^)^ and its transcriptional partner *Lef1*,^(^ ^35^ ^)^ or overexpression of c‐myc, which is downstream of the β‐catenin pathway and inhibited by 1,25(OH)_2_D/VDR.^(^ ^36^, ^37^ ^)^ In general, these conditions show disruption of hair follicle cycling during catagen of the first adult hair cycle, show a separation of dermal papillae from the receding hair follicle during this time, show a loss of ability to reinitiate anagen after the first hair follicle cycle, and show a disruption of sonic hedgehog (Shh) signaling in the bulge, which appears critical for the reinitiation of anagen.^(^ ^28^, ^29^, ^38^, ^39^, ^40^, ^41^ ^)^ The dissociation of the dermal papilla from the hair bulb by the end of catagen is thought to account for the failure to initiate the subsequent anagen in both *Hr* mutant and *Vdr* null mice.^(^ ^29^, ^31^, ^34^ ^)^ Additional clues come from RNA‐seq data of keratinocyte stem cells from *Vdr* wild‐type and KO mice, which showed a number of genes that were upregulated in the KO cells, one of which was peroxisome proliferator‐activated receptor γ (*Pparg*).^(^ ^42^ ^)^ Haploinsufficiency of *Pparg* reduced the mRNA levels of this gene in *Vdr* null skin and restored hair growth in the *Vdr* KO.^(^ ^42^ ^)^ The concept here is VDR acts as a repressor blocking the expression of *Pparg* and other genes involved with the regulation of hair follicle cycling. A similar situation will be described for the hedgehog pathway and its role in skin cancer. The distal (epidermal) portion of the hair follicle including the sebaceous gland as well as the interfollicular epidermis are likewise impacted.^(^ ^29^, ^34^, ^43^, ^44^ ^)^ The large dermal cysts that develop with time contain markers of the differentiated interfollicular epidermis and sebaceous gland,^34^, ^43^, ^44^ ^)^ suggesting their origin from the distal portion of the hair follicle or epidermis, features similar to that found in *Hr* and *Ctnnb1* mutant animals.^(^ ^30^, ^34^, ^39^, ^40^ ^)^ We interpret these changes as due to altered cell fates in the stem cells that otherwise regulate not only the stem cells in the bulge controlling hair follicle cycling but also in the infundibulum of the hair follicle regulating sebaceous gland development and in the stratum basale regulating epidermal regeneration.^(^ ^45^ ^)^

The control of hair follicle development and cycling is complex,^(^ ^39^, ^40^ ^)^ and a large number of factors are implicated in this process. Disruption of canonical wnt signaling (*Ctnnb1* or *Lef1* mutations) disrupts both developmental and postnatal hair follicle cycling and is associated with loss of Shh expression.^(^ ^39^, ^40^ ^)^ Both *Hr* null^(^ ^46^ ^)^ and *Vdr* null mice show loss of Shh expression in the hair follicle. As will be discussed, this differs from the effect of *Vdr* deletion from the epidermis or keratinocytes in vitro. The *Hr* null mouse shows an increase in expression of the wnt inhibitor WISE,^(^ ^46^ ^)^ whereas we have shown a reduction of Wnt4 in the *Vdr* null hair follicle. Wnt4 is normally expressed in the matrix and precortex and would be expected to have an important role in interactions with the dermal papilla.^(^ ^47^ ^)^ Although we have not observed an obvious difference in β‐catenin mRNA levels, its protein levels and nuclear localization are reduced in the ORS keratinocytes and bulb of the hair follicle in the *Vdr* null mouse compared with wild‐type at the end of catagen. In other cells, VDR has been found to bind to β‐catenin directly, reducing its interaction with Tcf4 or Lef1 and so reducing the transcriptional activity of β‐catenin.^(^ ^48^, ^49^, ^50^ ^)^ On the other hand, Lef1 binds to VDR in its N terminal region independent of β‐catenin but nevertheless important for wnt signaling.^(^ ^41^ ^)^ Therefore, function of β‐catenin rather than expression may be altered in *Vdr* null hair follicles. As mentioned, VDR binds to β‐catenin, repressing its transcriptional activity, although this binding is stimulated by 1,25(OH)_2_D_3_.^(^ ^48^ ^)^ However, some binding of VDR to β‐catenin occurs in the absence of ligand,^(^ ^44^ ^)^ and in cells such as keratinocytes with a lot of β‐catenin, this may suffice to regulate transcription. The C terminal region of β‐catenin (aas 671–781) and the AF‐2 (C terminus) domain of VDR are required for this binding.^(^ ^48^ ^)^ Mutation at L417S of the *Vdr* in mice blocks this binding as well as *Vdr* transcriptional activity.^(^ ^49^ ^)^ Surprisingly, the mutation E420Q, which also prevents transcriptional activity, is still capable of binding β‐catenin, and in the presence of high levels of β‐catenin, some transcriptional activity can be achieved.^(^ ^49^ ^)^ This mutation causes rickets but not alopecia, suggesting that the ability of VDR to bind β‐catenin possibly in the absence of 1,25(OH)_2_D may enable it to support hair follicle cycling.

We and others have found VDR and Hr expressed in the nuclei of keratinocytes in the stratum basale, ORS, and matrix of the hair follicle.^(^ ^31^, ^45^, ^46^, ^51^, ^52^, ^53^ ^)^ Hr has characteristics of a coregulator in that it resides in the nucleus; its structure contains a nuclear localization signal, a putative zinc finger, and three LXXLL motifs^(^ ^54^ ^)^ like that found in coactivators that interact with nuclear hormone receptors such as VDR in the presence of ligand as well as ΦXXΦΦ motifs (Φ = hydrophobic amino acid) similar to regions in corepressors like SMRT and NCoR responsible for the binding of these corepressors to nuclear hormone receptors in the absence of ligand. In the brain, Hr has been suggested as a corepressor of the thyroid receptor (THRb) in that Hr can bind to THRb and inhibit its transcriptional activity.^(^ ^55^ ^)^ However, Hr does not appear to regulate thyroid hormone action in the keratinocyte.^(^ ^56^ ^)^ Rather, VDR appears to be the target in keratinocytes.^(^ ^57^ ^)^ Hsieh and colleagues^(^ ^51^ ^)^ demonstrated that Hr could bind to VDR in COS cells. They noted that Hr bound to VDR in the same region predicted for corepressor binding, and different from the C‐terminal region to which coactivators bind. The region of Hr responsible for VDR binding contains one LXXLL motif but also a ΦXXΦΦ motif, and only mutations in the ΦXXΦΦ motif altered binding to VDR.^(^ ^51^ ^)^ However, when we tested both motifs separately for their binding to VDR, both did with comparable affinity.^(^ ^58^ ^)^ Neither motif was substantially affected by 1,25(OH)_2_D, consistent with the findings of others that Hr/VDR binding is ligand independent.^(^ ^51^ ^)^ Binding of Hr to VDR correlated with inhibition of 1,25(OH)_2_D_3_ stimulation of a Cyp24a1 (24‐hydroxylase) promoter construct containing the VDRE of this vitamin D target gene.^(^ ^57^ ^)^ We have shown that the endogenous VDR binds to endogenous Hr in keratinocytes.^(^ ^57^ ^)^ Overexpression of Hr blocks the ability of 1,25(OH)_2_D_3_ to induce differentiation markers in keratinocytes, whereas inhibition of Hr expression enhances the stimulation by 1,25(OH)_2_D_3_ of these markers.^(^ ^57^ ^)^ The *Hr* null animal demonstrates upregulation of differentiation markers in the epidermis^(^ ^30^ ^)^ (the opposite to that found in the *Vdr* or *Cyp27b1* null animal) consistent with a corepressor role for Hr in vitamin D‐regulated epidermal differentiation. This difference between VDR and Hr in the epidermis emphasizes that the signaling pathways in epidermal differentiation are not the same as in hair follicle cycling and can be just the opposite. Antibodies to Hr enhance the binding of VDR to VDREs in vitamin D target genes in gel retardation assays,^(^ ^57^ ^)^ suggesting that Hr binding to VDR blocks its binding to VDREs. Using the chromatin immunoprecipitation assay (ChIP), we noted that 1,25(OH)_2_D_3_ displaced Hr from the VDREs tested, whereas 1,25(OH)_2_D_3_ recruited the coactivators DRIP205 (aka Med 1) and SRC3 to these same VDREs.^(^ ^57^ ^)^

These data indicate that Hr and β‐catenin can alter VDR transcriptional activity. But the question remains how this translates into regulation of hair follicle cycling or epidermal differentiation that is independent of 1,25(OH)_2_D_3_. That question remains.

## Skin Cancer

The ligand‐independent actions of VDR acting as a tumor suppressor in skin are less clear than that in hair follicle cycling. However, two publications demonstrate that *Vdr* null mice develop skin tumors after UVB radiation or administration of the chemical carcinogen 7,12 dimethylbenzanthracene (DMBA) but mice lacking Cyp27b1 and so lacking 1,25(OH)_2_D production do not.^(^ ^59^, ^60^ ^)^ That said, discerning mechanisms that are not also influenced by 1,25(OH)_2_D have not been performed in a consistent fashion.

The potential for vitamin D signaling as protection against epidermal tumor formation was demonstrated when Zinser and colleagues^(^ ^61^ ^)^ observed that 85% of the *Vdr* null mice but none of the controls developed skin tumors within 2 months of DMBA administration. These were primarily papillomas. These results have been confirmed using topical administration of DMBA/TPA.^(^ ^62^ ^)^ However, although only papillomas were observed in the *Vdr* null mice, *Rxrα* null mice developed both basal cell carcinomas (BCC) and squamous cell carcinomas (SCC).^(^ ^62^ ^)^ Subsequently, Ellison and colleagues^(^ ^59^ ^)^ and our own group^(^ ^63^ ^)^demonstrated that *Vdr* null mice were also more susceptible to tumor formation after UVB, and many of the tumors were SCC and BCC, but *Cyp27b1* KO mice were not. The appearance of BCC in these studies was initially surprising because the typical malignancy induced in mouse skin by UVR, ionizing radiation, or chemical carcinogens is SCC, not BCC.^(^ ^64^ ^)^ Given that BCC generally result from increased hedgehog (Hh) signaling^(^ ^65^ ^)^ and that lack of VDR results in BCC when β‐catenin signaling is increased,^(^ ^66^ ^)^ we became interested in the relationship between vitamin D, Hh, and β‐catenin signaling in tumor suppression. We also discovered that VDR regulated the expression of long non‐coding RNAs (lncRNAs) such that in the *Vdr* null mouse epidermis the balance between oncogenic and tumor suppressor lncRNAs was shifted to oncogenic species.^(^ ^67^ ^)^ Additionally, we^(^ ^63^ ^)^ noted a reduction in clearance in cyclobutane pyrimidine dimers (CPD) after UVB exposure of the skin of *Vdr* null mice, suggesting that disruption of DNA damage repair was playing a role in tumor susceptibility in these mice. Hr may also play a role in epidermal carcinogenesis as it does in hair follicle cycling in that mice with mutations in *Hr* are also quite susceptible to UVB‐induced skin cancer,^(^ ^68^, ^69^ ^)^ but Hr has not been studied in its relationship to VDR during skin cancer protection. In what follows, I will examine potential mechanisms and pathways within those mechanisms for their contribution to the role of VDR as a tumor suppressor, including regulation of proliferation and differentiation with particular attention to the Hh and wnt/β‐catenin pathways, long non‐coding RNAs, and DNA damage repair.

### Vitamin D regulation of epidermal proliferation and differentiation

1,25(OH)_2_D increases essentially every step of the differentiation process in the epidermis,^(^ ^70^, ^71^, ^72^, ^73^, ^74^, ^75^ ^)^ while inhibiting proliferation at least at concentrations above 1 nM. These actions complement those of calcium, but calcium at least in vitro does not require 1,25(OH)_2_D as keratinocyte differentiation is readily induced by calcium in serum‐free media.^(^ ^76^ ^)^ However, the calcium response is enhanced by 1,25(OH)_2_D via its induction of the calcium‐sensing receptor (CaSR),^(^ ^77^, ^78^ ^)^ which along with induction of the phospholipase C enzymes^(^ ^79^, ^80^, ^81^ ^)^ regulates intracellular calcium and other signaling molecules critical for the differentiation process. On the other hand, at least some of these 1,25(OH)_2_D‐independent actions of calcium require VDR. An example of this is the effect of calcium on the translocation of E‐cadherin to the membrane after calcium administration, which is blunted in keratinocytes lacking the VDR.^(^ ^82^ ^)^ Thus, calcium stimulation of keratinocyte differentiation does not appear to depend on 1,25(OH)_2_D, but at least some of its actions require VDR in the absence of 1,25(OH)_2_D.

Two pathways important in vitamin D signaling in the epidermis with respect to proliferation and differentiation that we believe underlie the predisposition of the *Vdr* null mouse to tumor formation and that are at least partially regulated by VDR in a 1,25(OH)_2_D‐independent fashion are the Hh and wnt/β‐catenin pathways, pathways likely not coincidentally also playing important roles in hair follicle cycling.

#### The hedgehog pathway

In the skin, SHH is the ligand for patched (PTCH) 1, a 12‐transmembrane domain protein that in the absence of SHH inhibits the function of another membrane protein smoothened (SMO). SMO in turn maintains a family of transcription factors, GLI1 and GLI2 in particular, in the cytoplasm bound to suppressor of fused (SUFU).^(^ ^83^, ^84^ ^)^ When SHH binds to PTCH 1, the inhibition of SMO is relaxed, GLI1 and 2 are released from SUFU, and they move into the nucleus, where they initiate transcription of a number of factors, including each other as well as PTCH 1, the anti‐apoptotic factor BCL2, cyclins D1 and D2, E2F1, and CDC45 (all of which promote proliferation), while suppressing genes associated with keratinocyte differentiation, such as the keratins K1and K10, involucrin, loricrin, and the VDR.^(^ ^85^, ^86^, ^87^, ^88^, ^89^ ^)^

The appearance of BCC is characteristic of tumors formed when Hh signaling is activated,^(^ ^90^ ^)^ although activation of Hh signaling also predisposes to UVR‐induced SCC formation.^(^ ^91^ ^)^ *Vdr* null animals overexpress elements of the Hh signaling pathway in their epidermis and the epidermal portion (utricles) of the hair follicles.^(^ ^63^ ^)^ Moreover, 1,25(OH)_2_D suppresses the expression of all elements of the Hh pathway in a dose‐dependent fashion that requires the VDR^(^ ^63^, ^92^ ^)^ and reduces tumor growth in *Ptch* 1 null mice. On the other hand, VDR in the absence of 1,25(OH)_2_D also has a suppressor effect on this pathway, in that keratinocytes in which the VDR is knocked down overexpress both SHH and GLI1.^(^ ^63^ ^)^ The promoters of SHH and GLI1 have binding sites for VDR^(^ ^41^ ^)^ suggesting that the effects of VDR on these genes is direct.

#### The Wnt/β‐catenin pathway

Wnt signaling via activation of β‐catenin has a complex role in VDR function. In the canonical pathway, the receptor for Wnt ligands is a family of seven‐transmembrane Frizzled receptors and an LRP5 or LRP6 coreceptor. When Wnt binds to this complex, disheveled (Dvl) is phosphorylated, resulting in disruption of the axin/APC complex and inhibition of glycogen synthase kinase 3β (GSK‐3β). In the basal state, GSK‐3β phosphorylates the serine(s) within exon 3 of β‐catenin, resulting in its degradation by the E3 ubiquitin ligase. Wnt signaling, by blocking this phosphorylation, increases the availability of β‐catenin in the nucleus, where it binds to transcription factors of the T‐cell factor (TCF) and lymphoid enhancer factor (LEF) families to promote expression of genes such as cyclin D1 and c‐myc,^(^ ^93^ ^)^ important for proliferation. β‐catenin also forms part of the adherens junction complex with E‐cadherin, where it plays an important role in keratinocyte differentiation.^(^ ^94^ ^)^ Tyrosine phosphorylation of E‐cadherin, as occurs after calcium administration to keratinocytes, promotes the binding of β‐catenin and other catenins to the adherens junction complex^(^ ^94^, ^95^ ^)^ making it less available for transcriptional activity. As noted earlier, calcium and 1,25(OH)_2_D increase E‐cadherin expression and its membrane localization in a VDR‐dependent fashion,^(^ ^48^ ^)^ although as noted this action of calcium does not require 1,25(OH)_2_D. Overexpression and/or activating mutations in the β‐catenin pathway lead to skin tumors, in this case pilomatricomas or trichofolliculomas (hair follicle tumors).^(^ ^96^, ^97^, ^98^ ^)^ VDR binds to β‐catenin and reduces the transcriptional activity of β‐catenin in a 1,25(OH)_2_D‐dependent fashion.^(^ ^50^ ^)^ On the other hand, binding of β‐catenin to VDR in its AF‐2 domain enhances the 1,25(OH)_2_D**‐**dependent transcriptional activity of VDR.^(^ ^49^ ^)^ Palmer and colleagues^(^ ^66^ ^)^ evaluated the interaction between VDR and β‐catenin in transcriptional regulation in keratinocytes and identified putative response elements for VDR and β‐catenin/LEF in a number of genes. These interactions were either positive or negative, depending on the gene being evaluated. The hypothesis put forward is that genes in which the interaction was positive (ie, stimulated transcription) benefited from β‐catenin acting as a coactivator for VDR on VDREs, whereas in situations where the interaction was negative (ie, suppression of transcription) VDR prevented β‐catenin from binding to TCF/LEF required for transcription in those genes. We^(^ ^99^ ^)^ have found in keratinocytes that knockdown of VDR reduces E‐cadherin expression and formation of the β‐catenin/E‐cadherin membrane complex, resulting in increased β‐catenin transcriptional activity, whereas 1,25(OH)_2_D administration has the opposite effect. This was associated with increased (with VDR knockdown) or decreased (with 1,25(OH)_2_D administration) keratinocyte proliferation and cyclin D1 expression, providing evidence that VDR in the absence of 1,25(OH)_2_D might be acting to suppress this β‐catenin responsive gene.

##### Long non‐coding RNAs (LncRNAs)

Only about 2% of the genome is actively transcribed and translated into proteins, while a much larger percentage of the genome is actively transcribed without protein coding potential.^(^ ^100^ ^)^ These non‐coding transcripts can be broadly categorized into short and long non‐coding RNAs. The arbitrary size delineation is at 200 bases in length: small non‐coding RNAs are less than 200 bases, whereas lncRNAs are endogenous cellular RNAs larger than 200 bases and can even be greater than 100 kb in length.^(^ ^101^ ^)^ LncRNAs account for 80% of the transcriptome;^(^ ^100^ ^)^ they are spliced and contain polyadenylation signals, much like messenger RNAs.^(^ ^102^ ^)^ LncRNAs are expressed across all mammalian genomes and have emerged as master regulators of embryonic pluripotency, differentiation, and body axis patterning, promoting developmental transitions^(^ ^102^, ^103^ ^)^ and regulating histone modifications, hence influencing the epigenetic programs of the transcriptome.^(^ ^104^ ^)^ A number of these lncRNAs when aberrantly expressed are associated with cancers. We explored the potential role of lncRNAs in VDR protection against skin tumor formation by profiling 90 well‐annotated mouse lncRNAs from mouse keratinocytes cultured in vitro and mouse epidermis from epidermal‐specific *VDR* null mice and their normal littermates.^(^ ^67^, ^105^ ^)^ We found that several well‐known oncogenes, including *H19*, *HOTTIP*, and *Nespas*, are significantly increased, whereas tumor suppressor lncRNAs (*Kcnq1ot1*, *lincRNA‐p21*) were attenuated in VDR deleted keratinocytes. These were serum‐free cultures indicating a 1,25(OH)_2_D independent action of VDR on these lncRNAs. A similar pattern of lncRNA‐expression profiling was observed in the epidermis of epidermal‐specific *Vdr* null mice versus control littermates. In addition to the altered lncRNAs (*H19*, *HOTTIP*, *Nespase*, *Kcnq1ot1*, *lincRNA‐p21*) in *VDR* deleted human cultured keratinocytes, there was an increase in other oncogenes (*mHOTAIR*, *Malat1*, and *SRA*) and a decrease in other tumor suppressors (*Foxn2‐as*, *Gtl2‐as*, *H19‐as*) in *Vdr* null mouse epidermis. However, we have not documented direct effects of VDR on the genes expressing these lncRNAs or demonstrated their regulation by 1,25(OH)_2_D.

##### Vitamin D regulation of the DNA damage response

DNA damage response (DDR) is the means by which UVR and chemical‐induced DNA damage is prevented from producing fixed DNA mutations.^(^ ^106^ ^)^ DDR involves a cascade of damage recognition, repair, and signal transduction that coordinates the response of the cell to DNA damage. DDR activates checkpoints that delay the cell cycle, provides time for repair, and directs damaged cells into senescent or apoptotic pathways. DDR involves a number of components, is well orchestrated, tightly controlled, and highly accurate in normal primary cells such that the spontaneous mutation rate is very low, and changes in copy number are negligible.^(^ ^107^, ^108^, ^109^ ^)^ As noted earlier, UVB causes CPD and 6‐4PP formation, which are bulky adducts that block the movement of replicative DNA polymerase, a high‐fidelity enzyme, with a shift to trans lesion synthesis by lower‐fidelity DNA polymerases.^(^ ^110^ ^)^ Moreover, CPDs, if they occur in promoter regions, can block the binding of transcription factors.^(^ ^111^ ^)^ With malignant transformation, DDR becomes less controlled, and mutation rates and copy number abnormalities increase by orders of magnitude.^(^ ^107^, ^108^, ^112^, ^113^ ^)^ Nucleotide excision repair (NER) is the principal means by which UVR damage is repaired, enabling repair before DNA replication begins. This is important as NER plays a major role in reducing the amount of damage that becomes fixed as mutations during replication.^(^ ^114^, ^115^, ^116^ ^)^ During NER, the DNA damage is recognized by XPC acting in a complex with hRAD23B supported in some cases by the DNA damage‐binding protein DDB1 and 2,^(^ ^117^, ^118^ ^)^ the DNA is unwound around the lesion, and 30 base pair portions of DNA containing the lesion are excised by endonucleases such as XPF and XPG followed by fill‐in with DNA polymerases such as Pol δ,ε,κ.

The NER process has two main branches involving different mechanisms for the initial recognition of DNA damage^(^ ^119^ ^)^: transcription coupled repair (TCR), during which DNA polymerases stop replication at the site of the lesion until it is repaired,^(^ ^120^, ^121^, ^122^, ^123^, ^124^ ^)^ and global genomic repair (GGR), during which non‐transcribed regions of the genome are repaired.^(^ ^125^ ^)^ UVB increases VDR levels in keratinocytes.^(^ ^126^ ^)^ Keratinocytes in the epidermis of mice lacking *Vdr* are deficient in DDR as demonstrated by a reduced rate of clearing CPDs and 6,4PPs after UVB.^(^ ^126^ ^)^ Although 1,25(OH)_2_D can promote DNA damage repair at least in part through non‐genomic mechanisms, this action appears to be at least partially 1,25(OH)_2_D independent as epidermal explants from mice lacking *Vdr* likewise show this defect when evaluated in vitro in serum‐free media.^(^ ^126^ ^)^

## VDR as a Ligand‐Independent Transcriptional Regulator in Other Tissues

A number of studies have been performed comparing the phenotypes of mice lacking Cyp27b1 to those lacking Vdr or to mice in which the *Vdr* has been mutated to block 1,25(OH)_2_D binding in tissues other than the skin. These studies demonstrate that VDR, like other nuclear hormone receptors, can act as a regulator of gene transcription in the absence of its ligand.^(^ ^127^, ^128^ ^)^ Lee and colleagues^(^ ^129^, ^130^ ^)^ developed a knock‐in version of HVDRR in which the Vdr lacked the ability to bind 1,25(OH)_2_D (L233S mutation). In comparison to *Vdr* null mice, the mutant mice had higher PTH levels, comparable to *Cyp27b1* null mice, which they also examined. The *Cyp27b1* null mice also showed greater reductions in the intestinal expression of calcium transport genes, including *Trpv6* and *S100g* (calbindin 9 k) as well as *Atp2b1* (plasma membrane calcium ATPase) and *Slc30a10* (zinc transporter) than did the *Vdr* null mice, but other target genes did not differ in their expression between the two knockout models. In a somewhat different model in which the *Vdr* mutation in the ligand binding domain (L307H) eliminated 1,25(OH)_2_D binding but maintained binding to the 1,25(OH)_2_D analog Gemini (*Vdr* gem), Huet and colleagues^(^ ^131^ ^)^ found a more severe bone mineral phenotype in the *Vdr* gem mouse than in the *Vdr* null mouse, including a greater reduction in serum calcium and phosphate, increased alkaline phosphatase, and decreased bone mineral. They also observed more downregulated genes in microarray studies, including *Trpv6*, *Slc30a10*, and *Cyp24a1*, comparable to the results by Lee and colleagues^(^ ^130^ ^)^ in their model. Earlier studies comparing *Cyp27b1* and *Vdr* null mice on different calcium diets by Panda and colleagues^(^ ^132^ ^)^ likewise showed increased PTH and alkaline phosphatase levels and a more disorganized growth plate in the *Cyp27b1* null mouse compared with the *Vdr* null mouse. VDR can interact with other transcription factors that regulate its activity, including p53^(^ ^133^ ^)^ and Ets‐1,^(^ ^134^ ^)^ the latter showing ligand‐independent VDR stimulation of prolactin expression. The activation of FoxO requires its deacetylation and dephosphorylation, processes carried out by a complex of VDR/RXR in combination with Sirt1 and the catalytic subunit of the protein phosphatase 1. The recruitment of each to VDR is ligand independent.^(^ ^135^ ^)^ Thus, like other nuclear hormone receptors, ligand‐independent actions of VDR comprise a significant part of its mechanisms of action.

## Summary and Conclusions

Although most known actions of VDR require its ligand, 1,25(OH)_2_D, the unliganded receptor is also active (Table 1). The skin provides the best examples in that hair follicle cycling requires the VDR but not 1,25(OH)_2_D, and chemical‐ and UVB‐induced skin cancer occur in the *Vdr* null mouse but not in the *Cyp27b1* null mouse. With respect to hair follicle cycling, the ligand‐independent interactions between Hr, β‐catenin, and VDR appear of paramount importance. With respect to epidermal carcinogenesis, the suppressive influence of the unliganded VDR on the hedgehog and β‐catenin pathways appears to play a major role as does the promotion by VDR of the DNA damage repair process. Furthermore, comparisons between mice lacking Vdr and those lacking Cyp27b1 provide evidence that the unliganded VDR has an impact on parathyroid hormone production, bone growth, and expression of calcium transport and other genes in the intestine and elsewhere. No doubt, some of these actions may be indirect. However, VDR binding sites abound in the genome with remarkable cell‐to‐cell variation, and only a portion show dependence on 1,25(OH)_2_D for VDR binding to occur. This too shows remarkable cell specificity with the keratinocyte having the most overlap between VDR binding with and without 1,25(OH)_2_D and the intestine showing the least. There remains much to learn about the ligand‐independent actions of VDR, but as the field progresses, these actions must be considered to appreciate the full understanding of VDR mechanisms of action.

**Table 1:** VDR Actions at Least in Part Independent of 1,25(OH)2D

VDR action	Postulated mechanisms
Hair follicle cycling	Regulation of b‐catenin signaling
	Regulation of hedgehog signaling
	Regulation of hairless signaling
	Repression of PPARg expression
Skin cancer suppression	Regulation of b‐catenin signaling
	Regulation of hedgehog signaling
	Regulation of LncRNA expression
	Regulation of DNA damage response
Bone mineral response	Regulation of PTH production/secretion
	Regulation of calcium transport genes
	Regulation of bone growth and development

### Peer Review

The peer review history for this article is available at https://publons.com/publon/10.1002/jbm4.10578.
